# Sequential therapy of refractory metastatic pancreatic cancer with 5-FU/LV/irinotecan (FOLFIRI) vs. 5-FU/LV/oxaliplatin (OFF). The PANTHEON trial (AIO PAK 0116)

**DOI:** 10.1007/s00432-024-05827-x

**Published:** 2024-07-01

**Authors:** Dominik Paul Modest, Volker Heinemann, Philipp Schütt, Stefan Angermeier, Mike Haberkorn, Oliver Waidmann, Ullrich Graeven, Kai Wille, Volker Kunzmann, Larissa Henze, Christian Constantin, Maike de Wit, Claudio Denzlinger, Alexej Ballhausen, Annika Kurreck, Ivan Jelas, Annabel Helga Sophie Alig, Arndt Stahler, Sebastian Stintzing, Helmut Oettle

**Affiliations:** 1 Charité – Universitätsmedizin Berlin, Department of Hematology, Oncology, and Cancer Immunology, Freie Universität Berlin and Humboldt-Universität Zu Berlin, Augustenburger Platz 1, 13353 Berlin, Germany; 2 German Cancer Consortium (DKTK), German Cancer Research Centre (DKFZ), Heidelberg, Germany; 3 Department of Medicine III & Comprehensive Cancer Center, Hospital of the University (LMU), Munich, Germany; 4 Practice of Hematology and Oncology, Gütersloh, Germany; 5 Department of Gastroenterology, Hematology and Oncology, Hospital Ludwigsburg, Ludwigsburg, Germany; 6 ÜBAG MVZ Dr. Vehling-Kaiser GmbH, Landshut, Germany; 7 Centrum für Hämatologie und Onkologie Bethanien, Im Prüfling 17-19, 60389 Frankfurt, Germany; 8 Department of Hematology, Oncology and Gastroenterology, Klinken Maria Hilf GmbH, Mönchengladbach, Germany; 9 University Clinic for Haematology, Oncology, Haemostaseology and Palliative Care, Johannes Wesling Medical Center Minden, University of Bochum, Bochum, Germany; 10 Medical Clinic and Polyclinic II - IOT, University Hospital Würzburg, Würzburg, Germany; 11 Department of Medicine, Clinic III, Hematology, Oncology, Palliative Medicine, Rostock University Medical Center, Rostock, Germany; 12 Department for Oncology and Hematology, Clinical Center Lippe-Lemgo, Lemgo, Germany; 13 Department for Internal Medicine-Hematology and Oncology, VIVANTES Hospital Neukölln, Berlin, Germany; 14 Clinic for Internal Medicine 3, Marienhospital, Stuttgart, Germany; 15 Practice for Internal Medicine, Joint Practice and Day Clinic, Friedrichshafen, Germany

**Keywords:** Pancreatic cancer, Chemotherapy, Metastatic, FOLFIRI, Oxaliplatin, Fluoropyrimidine

## Abstract

**Purpose:**

In patients with metastatic pancreatic cancer, after failure of gemcitabine/nab-paclitaxel, this trial compares the efficacy of second-line therapy with FOLFIRI vs. OFF (1:1 randomisation) with cross-over to the vice-versa regimen as third-line therapy.

**Patients and Methods:**

The primary endpoint was PFS (progression-free survival: time from randomization until progression or death) of second-line therapy. The trial aimed to demonstrate non-inferiority of FOLFIRI vs OFF (non-inferiority margin of a hazard ratio (HR) of 1.5, power of 80% and a significance level of 5%, 196 events needed). Secondary endpoints included overall survival (OS), progression-free survival of third-line therapy and safety. The trial is registered with EudraCT Nr. 2016–004640-11.

**Results:**

The trial was terminated with 60 evaluable (37 with FOLFIRI, 23 with OFF) patients due to insufficient recruitment. PFS of second-line therapy was 2.4 (95% CI 2.3–2.6) months with FOLFIRI vs 2.4 (95% CI 2.2–2.7) months with OFF (HR: 0.80, 95% CI 0.45–1.42, P = 0.43). OS was comparable between the arms (HR: 0.95, 95% CI 0.54–1.66), P = 0.84). Only 4 out of 28 (14%) patients receiving third-line therapy achieved a disease control (partial remission or stable disease). Both second-line regimens were well tolerated without new or unexpected safety signals being observed.

**Conclusion:**

The exploratory analysis of this early terminated trial suggests that FOLFIRI and OFF have similar efficacy ant toxicity as second-line therapy of PDAC after failure of gemcitabine/nab-paclitaxel. Third-line therapy regardless of regimen does not provide satisfactory efficacy in this sequential treatment algorithm.

**Supplementary Information:**

The online version contains supplementary material available at 10.1007/s00432-024-05827-x.

## Introduction

Systemic therapy of advanced or metastatic pancreatic ductal adenocarcinomas (PDAC) usually involves doublet or triplet chemotherapy regimens (Conroy et al. 2011; Hoff et al. 2013). Of those the combination of 5-FU, folinic acid, oxaliplatin and irinotecan (FOLFIRINOX) represents a standard of care for the treatment of an advanced and/or metastatic systemic disease (Conroy et al. 2011; Conroy et al. 2018). If FOLFIRINOX is not an option for patients in need for systemic therapy of metastatic PDAC, the combination of gemcitabine plus nab-paclitaxel can be considered as standard of care (Hoff et al. 2013). If therapy is started with gemcitabine/nab-paclitaxel, the continuum of therapy beyond this decision is not entirely clear. Currently, two doublet-chemotherapy options might be considered, containing the drugs FOLFIRINOX consists of (Yoo et al. 2009): either 5-FU, folinic acid, oxaliplatin (OFF) (Oettle et al. 2014) or a combination of (nano-liposomal) irinotecan and 5-FU plus folinic acid (Nal-IRI-FF or FOLFIRI) (Ueno et al. 2020; Wang-Gillam et al. 2016). In clinical practice, the actual choice of therapy might involve existing toxicities and patients’ preferences- taking into account the differential profiles of these regimens concerning adverse events.

Beyond second-line therapy, no systemic therapy is established yet (Ducreux et al. 2015), although the remaining options (FOLFIRI or OFF, depending on the choice of second-line therapy) might be available if therapy is started with gemcitabine/nab-paclitaxel.

The PANTHEON trial was designed to compare the efficacy and tolerability of two established second-line therapy options (FOLFIRI vs. OFF) following gemcitabine/nab-paclitaxel as first-line therapy for metastatic pancreatic cancer. Furthermore, the trial explored the outcome of third-line therapy with the respective cross-over regimen.

## Methods

### Patients

Main inclusion criteria included: Unresectable adenocarcinoma of the pancreas previously treated in the palliative setting with gemcitabine and nab-paclitaxel, adequately documented recurrence and disease status after/with first-line therapy (best response, duration of treatment, time to progression, pre-existing polyneuropathy and other side effects), radiologically confirmed disease progression during first-line therapy and measurable reference cancer site(s) as defined by RECIST 1.1, and ECOG performance status 0–2.

### Design of the trial and endpoints

**Fig. 1:**
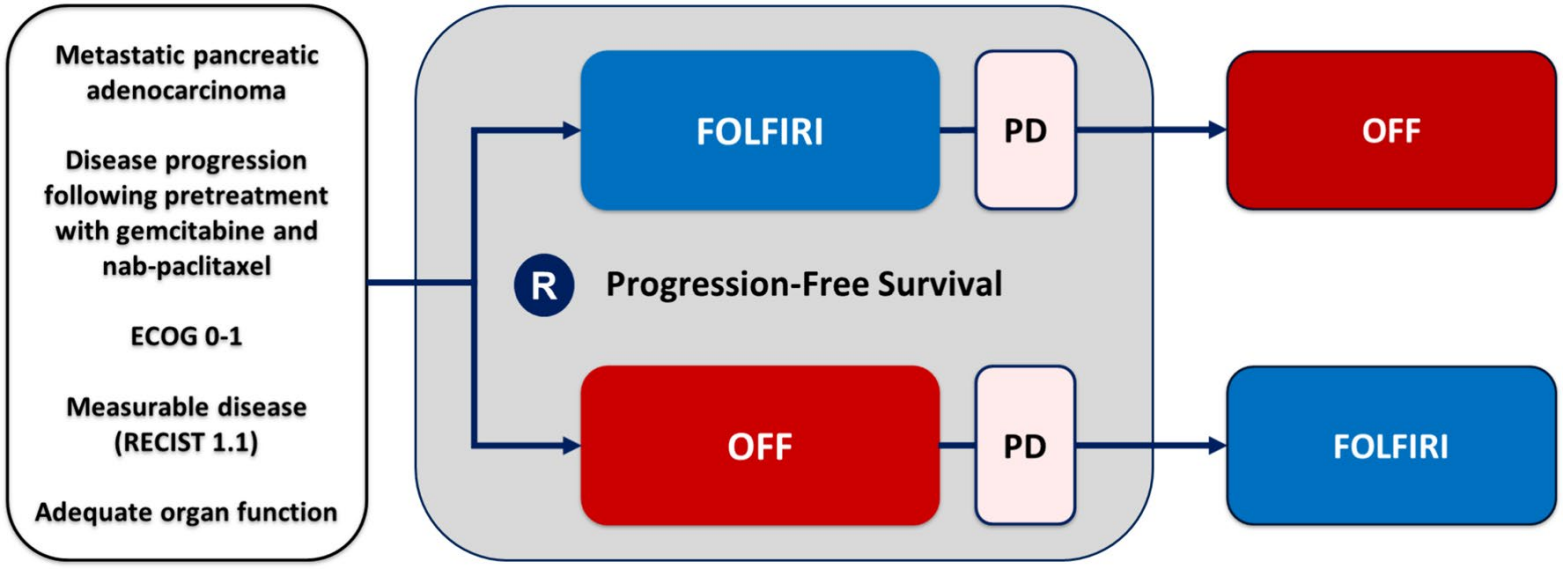
Study design. FOLFIRI denotes irinotecan, 5-fluorouracil and folinic acid. OFF denotes 5-fluorouracil, folinic acid and oxaliplatin

The trial was designed by HO within the Arbeitsgemeinschaft Internistische Onkologie (AIO) working group of pancreatic cancer. The trial randomized patients in a 1:1 ratio into FOLFIRI vs OFF. After failure of these second-line regimens, patients fit to receive a third-line therapy crossed over to the respective other regimen (please refer to Fig. 1). Randomization into treatment arms was organized centrally using permuted blocks. Randomization was stratified by ECOG performance status 0–1 vs. 2, pre-existing neuropathy grade 0–1 vs. 2, progression-free survival of first-line therapy < six months vs. ≥ six months.

The primary endpoint was progression-free survival (PFS) of the randomized second-line therapy (FOLFIRI or OFF) as assessed as by the local investigators as time from enrolment to disease progression according to RECIST 1.1 or death. The aim of the trial was to demonstrate non-inferiority of FOLFIRI compared to OFF. The statistical design assumed a PFS with OFF of 3.0 months. A considerably shorter PFS (i.e. 2.0 months) was considered as an inferior outcome. With this non-inferiority margin of a hazard ratio (HR) of 1.5 (3/2 months), a power of 80% and a one-sided significance level of 2.5%, it was intended to recruit 204 patients to observe 196 events. Secondary and exploratory endpoints included overall survival (within study, measured from randomization), overall survival of the complete treatment sequence (including previous first-line therapy), safety (AEs/SAEs), progression-free survival during third line therapy (PFS3 = administration of first dose of third-line therapy to progression or death), Time to Failure of Strategy (TTFS = time from randomization to permanent study treatment discontinuation (second or third-line), as well as objective response rates according to RECIST 1.1. The trial is registered with EudraCT Nr. 2016–004640-11.

### Statistical analysis

The pre-planned analysis of the primary endpoint (PFS) was prespecified to be analysed in a per-protocol (PP) population, defined as: no violation of inclusion/exclusion criteria, patient received at least 1 dosing-cycle (full or reduced dosing) of chemotherapy, documentation of a tumor assessment after start of study treatment/randomization, absence of other major protocol violations such as wrong treatment received. All data recorded in the eCRF describing the sample, the efficacy and the safety are analyzed descriptively. Event-related data like progression-free or overall survival are estimated by the Kaplan–Meier method, and compared using the logrank test and/or a Cox regression. The primary hypothesis of the trial are evaluated by calculation of the one-sided 95% confidence interval for the hazard ratio in a univariate Cox model. In addition, a log-rank test on non-inferiority of the experimental arm is performed. All statistical analyses were done using Microsoft Excel, WinStat Version 2012.1.0.96 (R. Fitch Software, MA, USA), R Version 4.1.1 and SPSS 27, IBM. Data cut-off was April 28th 2022.

### Trial conduct

All patients provided written informed consent prior to screening in the trial. Patients were recruited in 19 centres in Germany. The protocol was approved by the responsible ethics committees of the participating centres. A contract research organisation (CROLLL GmbH, Nürnberg, Germany) was responsible for randomization, data management, monitoring and primary data analysis. The trial was conducted in accordance with the protocol and in compliance with the Declaration of Helsinki.

### Treatment

Biweekly FOLFIRI was irinotecan 180 mg/m^2^, folinic acid 400 mg/m^2^ (FA), 5-fluorouracil (5-FU): 400 mg/m^2^ bolus and 2400 mg/m^2^ (48 h). OFF was folinic acid 200 mg/m^2^ and 5-FU 2000 mg/m^2^ (24 h) on days 1, 8, 15, and 22 plus oxaliplatin 85 mg/m^2^ on days 8 and 22. OFF was repeated every 6 weeks.

### Assessments

The study protocol defined tumor assessment as computed tomography (CT) or magnetic resonance imaging according to standard of care. Following initial imaging (within 28 days before randomization), re-assessments were scheduled every ten weeks until disease progression or death. Assessments were performed according to RECIST 1.1. In case of end of study therapy without progressive disease, further regular assessments were recommended. Adverse events were documented according to the grading of the National Cancer Institute Common Terminology Criteria for Adverse Events (NCICTC-AE) from registration into the trial until the final study visit.

### Role of the funding source

The legal funder (sponsor) of the trial was the AIO-Studien-gGmbH, Berlin Germany. Bristol Myers Squibb GmbH & Co. KGaA (Celgene) supported the trial with a research grant to the AIO-Studien-gGmbH and had no role in the design conduct and analysis of the trial but reviewed the manuscript prior to journal submission. DPM and HO had full access to all study data and had the final responsibility for the decision to submit for publication.

## Results

### Patients and treatment

**Fig. 2:**
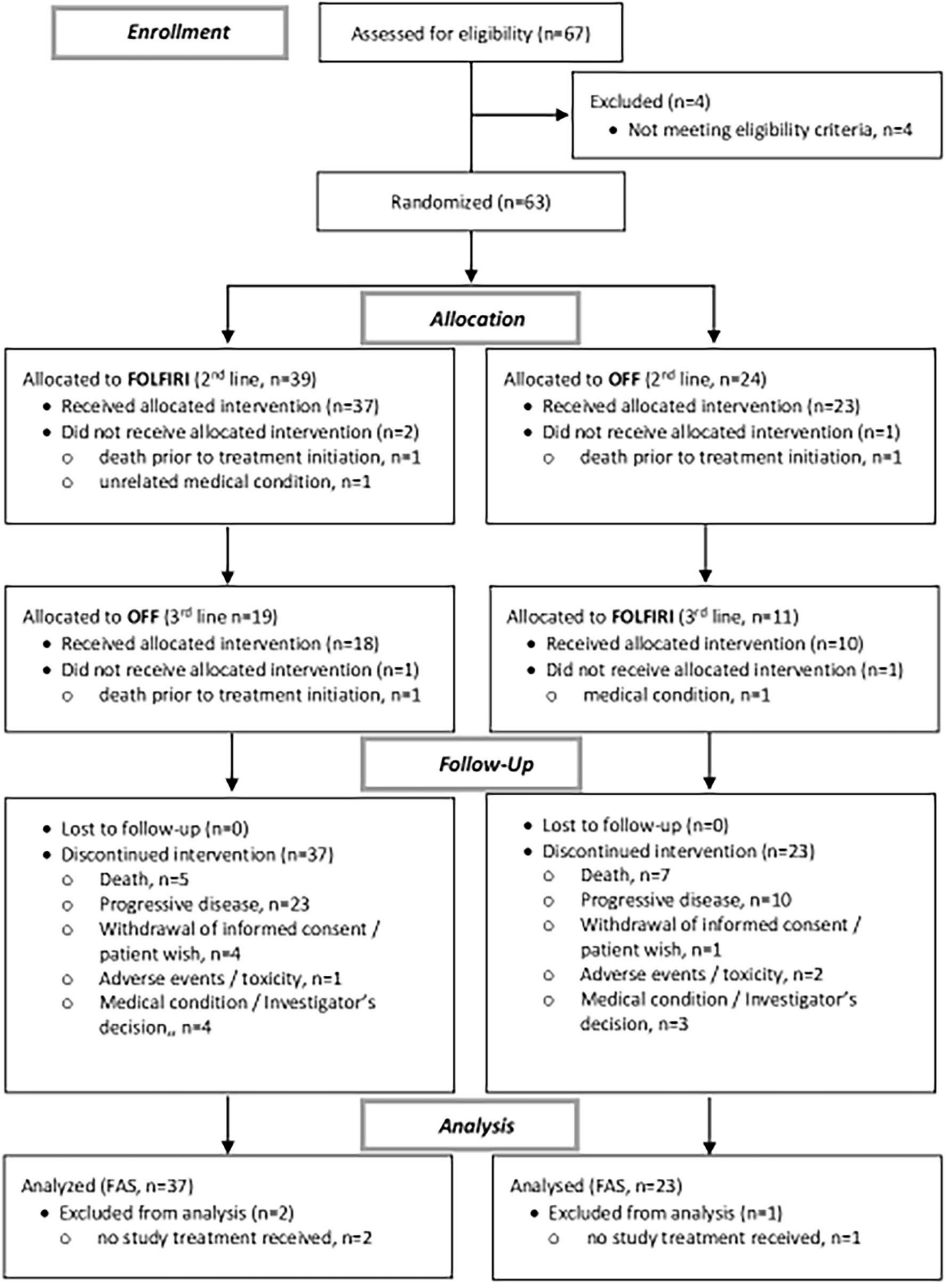
Consort diagram. FOLFIRI denotes irinotecan, 5-fluorouracil and folinic acid. OFF denotes 5-fluorouracil, folinic acid and oxaliplatin

The PANTHEON trial randomized 63 patients between January 2018 and December 2020. In December 2020 the study was stopped as the recruitment rate was insufficient to guarantee recruitment until the full sample size. Of the 63 recruited patients, 60 patients formed the full analysis set (37 patients in the FOLFIRI second-line arm and 23 patients in the OFF second-line therapy arm). Of note, the reduced sample size obtained within this study impacted balancing of prognostic groups and thus the balance of the randomisation as stratified permutated block randomization was incomplete. Third-line therapy was administered in 28 patients (18 patients received OFF following FOLFIRI and 10 patients the vice versa sequence)—please refer to Fig. 2 for details. Characteristics of patients and tumors are summarized in Table 1. Median follow-up of the full analysis set from start of the reported first-line therapy was 10.6 months (range 5.3–33.4 months).

**Table 1:** Patient and tumor characteristics at baseline

Characteristics	FOLFIRI arm, n = 37	OFF arm, n = 23
Sex, n (%)		
Female	19 (51.4)	14 (60.9)
Male	18 (48.6)	9 (39.1)
Age, median years (range)	68 (49–82)	65 (48–80)
ECOG performance status, n (%)		
0–1	36 (97.3)	21 (91.3)
2	1 (2.7)	2 (8.7)
Metastases present, n (%)		
No	3 (8.1)	0 (0)
Yes	34 (91.9)	23 (100)
Liver	25 (67.6)	19 (82.6)
Lung	11 (29.7)	6 (26.1)
Peritoneum	6 (16.2)	8 (34.8)
Lymph nodes	7 (18.9)	6 (26.1)
Bone	2 (5.4)	1 (4.3)
Adrenal glands	1 (2.7)	0 (0)
Brain	1 (2.7)	0 (0)
Other	1 (2.7)	5 (21.7)
Polyneuropathy grade (NCI CTCAE grade), n (%)		
0–1	29 (78.4)	20 (87.0)
2	8 (21.6)	3 (13.0)
Progression-free survival of prior 1st-line therapy, n (%)		
PFS < 6 months	17 (45.9)	9 (39.1)
PFS ≥ 6 months	20 (54.1)	14 (60.9)
Best response to prior 1st-line therapy, n (%)		
Complete remission (CR)	2 (5.6)	0 (0)
Partial remission (PR)	16 (44.4)	9 (39.1)
Stable disease (SD)	11 (30.6)	7 (30.4)
Progressive disease (PD)	8 (22.2)	7 (30.4)

FOLFIRI denotes irinotecan, 5-fluorouracil and folinic acid. OFF denotes oxaliplatin, 5-fluorouracil and folinic acid. ECOG = Eastern Cooperative Oncology Group. PFS (Conroy et al. 2011) = reported PFS of previous first-line therapy with gemcitabine/nab-paclitaxel

### Study treatment duration

Of the initial 37 patients who received FOLFIRI as second-line therapy, 18 patients were consecutively exposed to OFF (48.6%), while the vice versa sequence was observed in 10 of the initial 23 patients with second-line OFF therapy (43%) who consecutively received FOLFIRI as second-line therapy.

Median treatment duration of second-line therapy with FOLFIRI was 2.8 (range 0.9–12.2) months, while 2.5 (range 0.9–8.7) months were observed in the OFF second-line arm.

### Efficacy of study therapy, including the primary endpoint

**Fig. 3:**
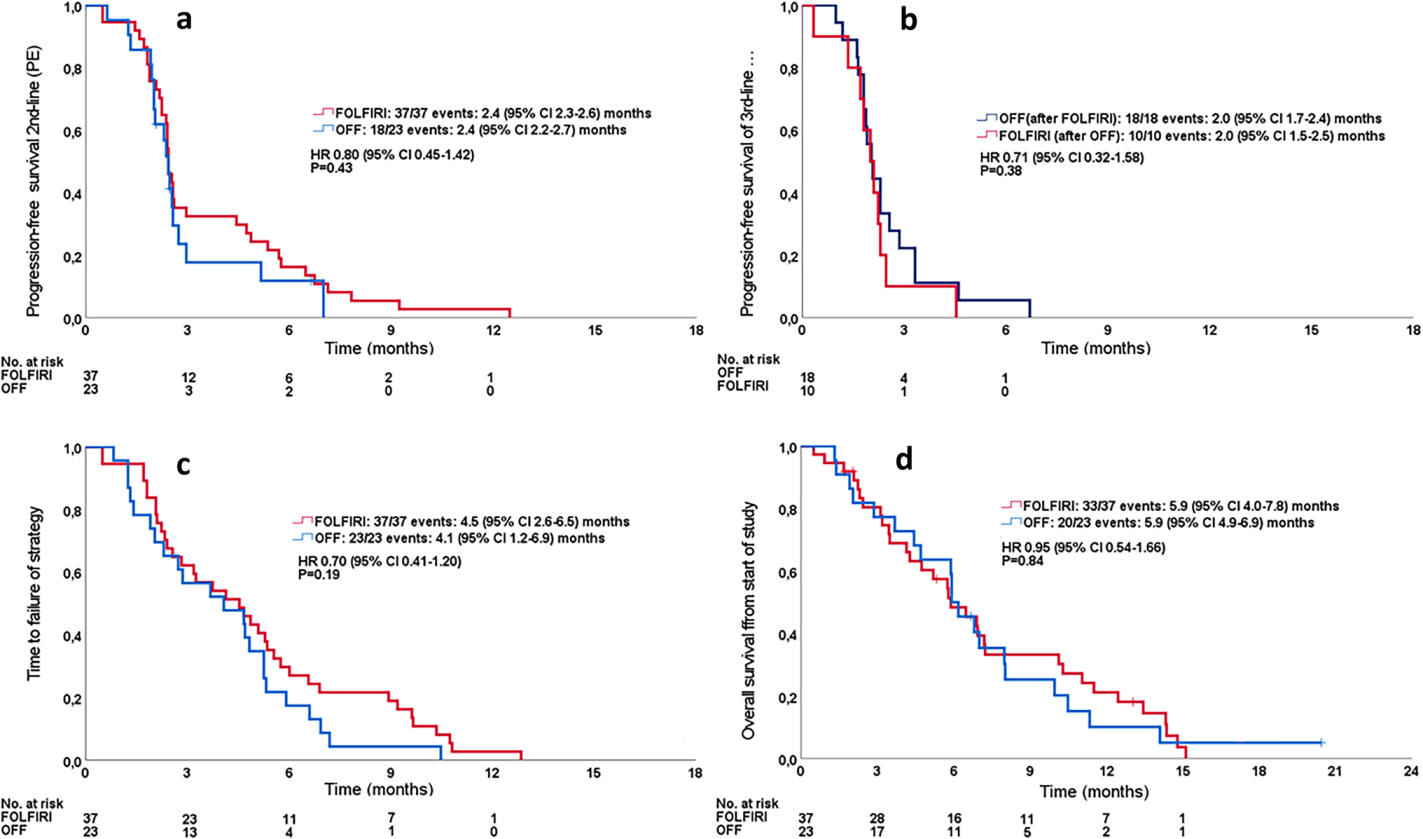
Kaplan–Meier estimates of the full analysis set for PFS, PFS3, TTFS and OS. Indicated hazard ratios derived from Cox regression testing. P-values derived from log rank tests. **A** Kaplan–Meier estimate of progression-free survival of second-line therapy (primary endpoint), **B** Kaplan–Meier estimate of progression-free survival of third-line therapy (secondary endpoint), **C** Kaplan–Meier estimate of time to failure of strategy (secondary endpoint), **D** Kaplan–Meier estimate of overall survival (secondary endpoint),

The primary endpoint of the trial (progression-free survival of second-line therapy) was analyzed with 55 of 60 possible events. The median PFS with FOLFIRI was 2.4 (95% CI 2.3–2.6) months, compared to 2.4 (95% CI 2.2–2.7) months with OFF (HR, 0.80; 95% CI 0.45 to 1.42; P = 0.45). The progression-free survival of third-line therapy was comparable in both arms. The estimation of time-to-failure of strategy (TTF) trended to be more favourable with FOLFIRI-OFF as compared to the reverse sequence (HR 0.70; 95% CI 0.41–1.20; P = 0.19). Overall survival was again similar in both arms of the trial when calculated from randomisation and also when including the previous first-line therapy; please refer to Fig. 3A–D for details.

In this trial (both arms of the trial analyzed together), ORR was 5.0% with second-line therapy and 3.6% with third-line therapy. Disease control rates were 25% in second-line therapy and 14.3% in third-line therapy, please refer to Table 2 for details.

**Table 2:** Objective response rates according to RECIST 1.1

RECIST evaluations, responses	Strategy 1	Strategy 2
Number, (%)	FOLFIRI 2nd-line (n = 37)	OFF 2nd-line (n = 23)
PR	3 (8.1)	0 (0.0)
SD	9 (24.3)	3 (13.0)
PD	18 (48.6)	12 (52.2)
n.a	7 (18.9)	8 (34.8)
ORR	3 (8.1)	0 (0.0)
DCR	12 (32.4)	3 (13.0)

Number, (%)	OFF 3rd-line (n = 18)	FOLFIRI 3rd-line (n = 10)
PR	1 (5.6)	0 (0.0)
SD	2 (11.1)	1 (10.0)
PD	14 (77.8)	7 (70.0)
n.a	1 (5.6)	2 (20.0)
ORR	1 (5.6)	0 (0.0)
DCR	3 (16.7)	1 (10.0)

*PR* partial remission, *SD* stable disease, *PD* progressive disease, *n.a.* not assessed, *ORR*  objective response rate (PR), *DCR * disease control rate (PR + SD)

### Toxicity and safety

During second-line therapy, 18 of 37 patients in the FOLFIRI arm (48.6%) and 15 of 23 (65.2%) in the OFF arm experienced a serious adverse event of any grade—with or without relation to the respective drugs.

In both second-line therapy arms, adverse events were most frequently observed in terms of gastrointestinal side effects, including nausea (45.9% and 34.8% of patients in the FOLFIRI and OFF arm, respectively, and diarrhoea (40.5% and 47.8% of patients, respectively). However, only a minority of these advents was grade 3–4 and of all gastrointestinal events, only diarrhoea and nausea were reported with more than 10% frequency of grade 3–4 (both in the OFF arm: 13%)- please refer to Table 3 for details.

**Table 3:** Grade 1–4 adverse events of interest with onset during second-line therapy

	FOLFIRI 2nd-line events, n | patients, n (%)		OFF 2nd-line events, n | patients, n (%)	
	Grade 1–4 (N = 37) %	Grade 3–4 (N = 37) %	Grade 1–4 (N = 23) %	Grade 3–4 (N = 23) %
Anemia	12 | 7 (18.9)	5 | 4 (10.8)	10 | 4 (17.4)	2 | 1 (4.3)
Leukopenia	5 | 4 (10.8)	1 | 1 (2.7)	6 | 3 (13)	1 | 1 (4.3)
Neutropenia	5 | 2 (5.4)	2 | 1 (2.7)	2 | 2 (8.7)	0 | 0 (0)
Diarrhoea	20 | 15 (40.5)	3 | 3 (8.1)	20 | 11 (47.8)	3 | 3 (13)
Nausea	25 | 17 (45.9)	1 | 1 (2.7)	21 | 8 (34.8)	3 | 3 (13)
Stomatitis	5 | 5 (13.5)	0 | 0 (0)	6 | 3 (13)	0 | 0 (0)
Vomiting	15 | 11 (29.7)	0 | 0 (0)	10 | 5 (21.7)	0 | 0 (0)
Cachexia	1 | 1 (2.7)	1 | 1 (2.7)	0 | 0 (0)	0 | 0 (0)
Decreased appetite	2 | 2 (5.4)	0 | 0 (0)	4 | 4 (17.4)	0 | 0 (0)
Fatigue	19 | 16 (43.2)	1 | 1 (2.7)	19 | 10 (43.5)	1 | 1 (4.3)
General physical health deterioration	6 | 5 (13.5)	3 | 2 (5.4)	2 | 2 (8.7)	1 | 1 (4.3)
Oedema peripheral	6 | 5 (13.5)	0 | 0 (0)	2 | 2 (8.7)	0 | 0 (0)
Pain	3 | 3 (8.1)	1 | 1 (2.7)	10 | 5 (21.7)	2 | 2 (8.7)
Pyrexia	5 | 3 (8.1)	0 | 0 (0)	7 | 6 (26.1)	0 | 0 (0)
Hypokalemia	9 | 4 (10.8)	3 | 2 (5.4)	4 | 3 (13)	2 | 2 (8.7)
Dizziness	0 | 0 (0)	0 | 0 (0)	6 | 3 (13)	0 | 0 (0)
Peripheral sensory neuropathy	6 | 4 (10.8)	1 | 1 (2.7)	10 | 6 (26.1)	2 | 1 (4.3)
Dyspnoea	7 | 7 (18.9)	0 | 0 (0)	2 | 2 (8.7)	0 | 0 (0)
Alopecia	5 | 5 (13.5)	0 | 0 (0)	0 | 0 (0)	0 | 0 (0)
Embolisms	2 | 2 (5.4)	1 | 1 (2.7)	0 | 0 (0)	0 | 0 (0)
Hypertension	2 | 2 (5.4)	1 | 1 (2.7)	0 | 0 (0)	0 | 0 (0)

Adverse event terms were derived from the case report forms. All events are reported irrespective of whether they were reported as related to study treatment. Grade according to NCI-CTCAE = National Cancer Institute Common Terminology Criteria for Adverse Event

## Discussion

The PANTHEON trial was designed with the objective to evaluate two different subsequent treatment strategies following first-line therapy with gemcitabine/nab-paclitaxel for metastatic pancreatic ductal adenocarcinoma. Unsatisfactory recruitment led to early termination of the trial. It might be speculated that this patient population is difficult to treat in study protocols and maybe also that homogenized criteria for the recruitment of a second-line study are hard to meet in metastatic pancreatic cancer.

Nevertheless, with 60 patients receiving active therapy in the trial, it might be concluded that both treatment option provided comparable efficacy in the trial.

The observed toxicities were more or less comparable in terms of frequency and reproduced the classic profiles of irinotecan vs. oxaliplatin-based treatment approaches. The overall frequency of oxaliplatin/irinotecan-related adverse events is in line with other gastrointestinal cancers receiving oxaliplatin- or irinotecan-based regimens in pretreated patient cohorts (Oettle et al. 2014; Wang-Gillam et al. 2016; Lamarca et al. 2021; Bennouna et al. 2013; Lorenzen et al. 2022).

The overall efficacy data (median survivals) in the PANTHEON trial, for both classic PFS of second-line therapy as well as in terms of OS appear similar to the respective pivotal trials which established OFF and the NAPOLI regimen (Oettle et al. 2014; Wang-Gillam et al. 2016), suggesting that the selection of patients in the PANTHEON trial was comparable to existing data. Given the efficacy of second-line therapy in our trial, it might be argued if this treatment option provides a clinically meaningful advantage to the majority of patients and in turn this treatment choice requires critical discussions with patients. Moreover, efficacy of combination therapy in third-line therapy was even less evident, suggesting that this treatment regimens do not have a sufficient rationale and should be considered with great caution. In turn, these perspectives question the overall strategy of the trial to explore a three-line strategy. Of course, if first-line therapy is done with FOLFIRINOX (or with nano-liposomal irinotecan) (Conroy et al. 2011), consecutive second-line treatment is usually gemcitabine-based, although there is no substantial evidence for this sequenceTaking these data into account and also the lack of efficacy of third-line therapy, it might be hypothesized that sequential therapy of metastatic pancreatic cancer should be planned within the first two lines of therapy. If first-line therapy is started with gemcitabine plus nab-paclitaxel, second-line therapy with either oxaliplatin- or irinotecan-based regimens might be considered the last potentially effective option. A novel option might be to integrate both regimens into the second-line therapy of PDAC by alternating OFF and FOLFIRI on the basis of promising reports from first-line trials (Carrato et al. 2022; Westphalen 2023).

A strength of this randomized trial might be the fact that it clearly suggests that initial therapy with gemcitabine plus nab-paclitaxel does not enable sufficient therapy across three lines.

Limits of the trial include the sample size and the lacking power to definitely demonstrate or reject the underlying hypothesis of the study protocol. Moreover, the randomization process by permuted blocks with three stratification criteria in a small sample size led to unbalanced numbers in the study arms which may reduce the validity of the results in the smaller (OFF) arm.

## Conclusion

This exploratory analysis of a terminated trial suggests that FOLFIRI and OFF have similar efficacy as second-line therapy of PDAC with comparable tolerability after failure of gemcitabine/nab-paclitaxel. The frequency of patients receiving crossover third-line therapy and the frequency of grade 3–5 events were also comparable. However, the sequence of three doublet regimes starting with gemcitabine/nab-paclitaxel does not lead to satisfactory efficacy in second-line and even less in the third-line of systemic therapy. These findings suggest that either irinotecan (or nano-liposomal irinotecan) or oxaliplatin cannot be successfully integrated into a treatment algorithm starting with gemcitabine/nab-paclitaxel.

### Supplementary Information

Below is the link to the electronic supplementary material.

Supplementary file1 (DOCX 13 KB)

## Data Availability

With respect to the clinical trial “Sequential therapy of metastatic pancreatic ductal adenocarcinoma (PDAC) after failure of gemcitabine plus nab-paclitaxel with either 5-FU/folinic acid (5FU/LV) plus oxaliplatin (OFF) followed by 5-FU/folinic acid plus irinotecan (FOLFIRI) or the reverse sequence.” (PANTHEON), AIO code AIO-PAK 0116, EudraCT-No. 2016-004640-11, AIO-Studien-gGmbH acting as th legal sponsor is committed to provide information about its results to researchers with the goal of facilitating scientific progress. All data shared must be anonymised to protect the privacy of the patients who participated in the trial, in accordance with applicable laws and regulations and in compliance with the International Council for Harmonisation and Good Clinical Practice (ICH/GCP).

## Declarations

### Prior presentation

IPart of the data of this manuscript have been presented as poster presentation at the ESMO Annual Meeting 2022.
