# Supplementary material for: Sequential therapy of refractory metastatic pancreatic cancer with 5-FU/LV/irinotecan (FOLFIRI) vs. 5-FU/LV/oxaliplatin (OFF). The PANTHEON trial (AIO PAK 0116)
Source: J Cancer Res Clin Oncol. 2024 Jul 1;150(7):332. doi: 10.1007/s00432-024-05827-x (PMC11217046; doi:10.1007/s00432-024-05827-x)
Supplement: Supplementary file 1 — Supplementary file1 (DOCX 13 KB) [file 432_2024_5827_MOESM1_ESM.docx]

| **Supplementary Table 1.** Representativeness of Study Participants | |
| --- | --- |
| Cancer | Metastatic pancreatic adenocarcinoma |
| Considerations related to: | |
| Sex | Pancreatic cancer affects both female and male patients with similar prevalence. The actual data for Germany (2020) report 20230 cases, of those 49.2% in female patients. |
| Age | The median age in patients with pancreatic cancer is 76 in female patients and 72 in male patients. |
| Race/ethnicity | There are no data on race/ethnicity and pancreatic cancer in the german cancer statistics. It is known that approximately 25-30% of the population in Germany has some kind of migration history background, with large numbers being reported for Turkey, Syria and the Ukraine as countries of origin. |
| Geography | In Germany, the incidence and mortality appear more or less similar in all counties. However, numerically lower numbers are observed in southern counties of Germany. |
| Other considerations | Given that the largest ethnic minorities in Germany represent a more or less homogeneous population, a disbalance of the small study population vs the overall population appears possible though not likely. |
| Overall representativeness of this study | The age distribution of our study is a little younger as compared to the population in the country, maybe owing to the selection for treatment eligibility.  The sex distribution in the small cohort appears balanced (33/60 female), also with respect to the overall distribution in the national statistics. No definitive conclusion concerning the distribution of race/ethnicity can be made based on available numbers. |
